# Dimorpholinium tetra­chlorido­cobaltate(II)

**DOI:** 10.1107/S1600536812035830

**Published:** 2012-08-23

**Authors:** Xing-Xing Cao, He-Long Cheng, Qing-Liu Feng, Li-Zhuang Chen

**Affiliations:** a School of Biology and Chemical Engineering, Jiangsu University of Science and Technology, Zhenjiang 212003, People’s Republic of China

## Abstract

In the title mol­ecular salt, (C_4_H_10_NO)_2_[CoCl_4_], the morpholinium cations adopt chair conformations and the tetra­chloridocobaltate(II) anion is significantly distorted from regular tetra­hedral geometry [Cl—Co—Cl = 102.183 (19)–117.59 (2)°]. The Co—Cl bond lengths for the chloride ions not accepting hydrogen bonds are significantly shorter than those for the chloride ions accepting such bonds. In the crystal, the components are linked by N—H⋯O and N—H⋯Cl and bifurcated N—H⋯(O,Cl) hydrogen bonds to generate (100) sheets.

## Related literature

For a phase transition in morpholinium tetra­fluoridoborate, see: Szklarz *et al.* (2009 ▶); Owczarek *et al.* (2008 ▶). For the structure of dimorpholinium penta­chloridoanti­monate(III), see: Chen (2009 ▶).
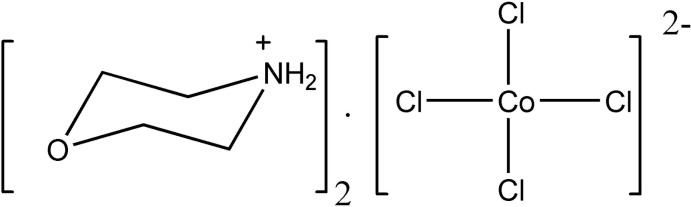


## Experimental

### 

#### Crystal data

(C_4_H_10_NO)_2_[CoCl_4_]
*M* *_r_* = 376.99
Monoclinic, 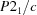

*a* = 6.5952 (13) Å
*b* = 13.696 (3) Å
*c* = 17.039 (3) Å
β = 92.930 (2)°
*V* = 1537.1 (5) Å^3^
*Z* = 4
Mo *K*α radiation
μ = 1.80 mm^−1^
*T* = 291 K
0.26 × 0.12 × 0.08 mm

#### Data collection

Rigaku SCXmini diffractometer
Absorption correction: multi-scan (*CrystalClear*; Rigaku, 2005 ▶) *T*_min_ = 0.90, *T*_max_ = 1.00
11708 measured reflections
2997 independent reflections
2761 reflections with *I* > 2σ(*I*)
*R*_int_ = 0.019

#### Refinement

*R*[*F* ^2^ > 2σ(*F* ^2^)] = 0.022
*wR*(*F* ^2^) = 0.074
*S* = 1.07
2997 reflections
154 parameters
H-atom parameters constrained
Δρ_max_ = 0.25 e Å^−3^
Δρ_min_ = −0.42 e Å^−3^

### 

Data collection: *CrystalClear* (Rigaku, 2005 ▶); cell refinement: *CrystalClear*; data reduction: *CrystalClear*; program(s) used to solve structure: *SHELXS97* (Sheldrick, 2008 ▶); program(s) used to refine structure: *SHELXL97* (Sheldrick, 2008 ▶); molecular graphics: *SHELXTL* (Sheldrick, 2008 ▶); software used to prepare material for publication: *SHELXL97*.

## Supplementary Material

Crystal structure: contains datablock(s) I, global. DOI: 10.1107/S1600536812035830/hb6916sup1.cif

Structure factors: contains datablock(s) I. DOI: 10.1107/S1600536812035830/hb6916Isup2.hkl

Additional supplementary materials:  crystallographic information; 3D view; checkCIF report

## Figures and Tables

**Table 1:** Selected bond lengths (Å)

Co1—Cl1	2.3029 (6)
Co1—Cl2	2.2720 (6)
Co1—Cl3	2.2455 (6)
Co1—Cl4	2.2811 (6)

**Table 2:** Hydrogen-bond geometry (Å, °)

*D*—H⋯*A*	*D*—H	H⋯*A*	*D*⋯*A*	*D*—H⋯*A*
N1—H1*C*⋯Cl1	0.90	2.39	3.1819 (15)	148
N1—H1*D*⋯O2^i^	0.90	1.97	2.8294 (19)	160
N2—H2*C*⋯O1^ii^	0.90	2.47	3.0577 (18)	123
N2—H2*C*⋯Cl4^iii^	0.90	2.57	3.3322 (15)	143
N2—H2*D*⋯Cl1^iv^	0.90	2.43	3.3003 (15)	164

Symmetry codes: (i) 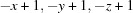; (ii) 
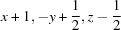
; (iii) 
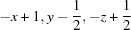
; (iv) 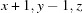.

**Fig. 1.**
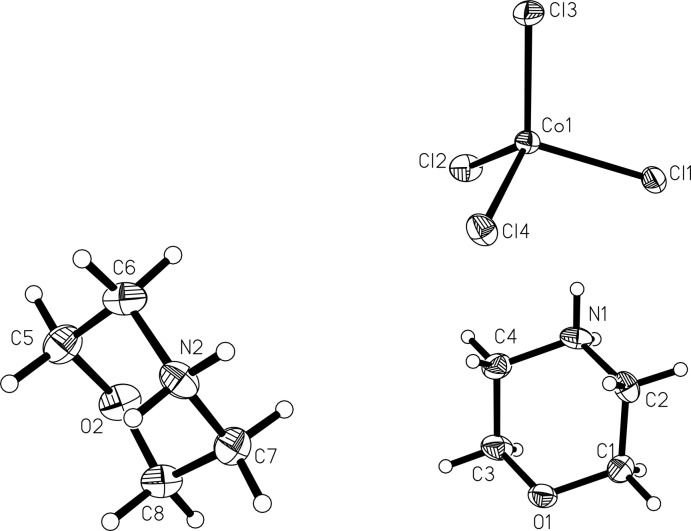
The asymmetric unit of the title compound. Displacement ellipsoids are drawn at the 30% probability level.

**Fig. 2.**
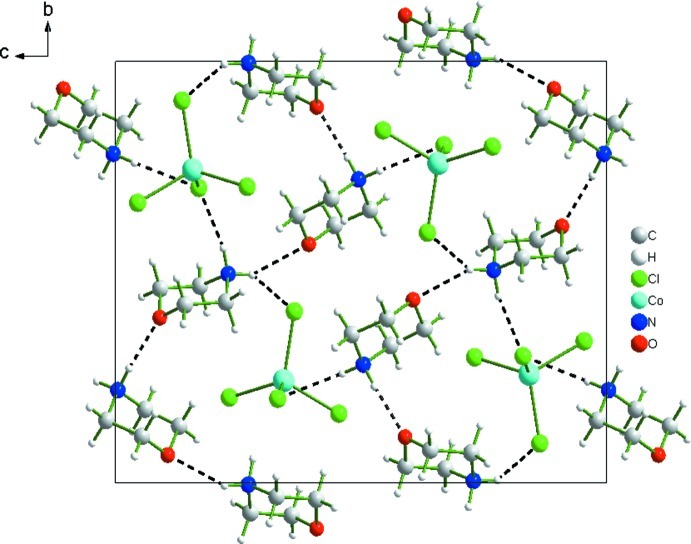
The packing viewed along the a axis. Hydrogen bonds are drawn as dashed lines

## supplementary crystallographic information

### Experimental

CoCl_2_ (2.37 g, 10 mmol), morpholine (1.01 g, 10 mmol) and 20% aqueous HCl in a molar ratio of 1:1:1 were mixed and dissolved in sufficient water by heating to 353 K forming a clear solution. The reaction mixture was cooled slowly to room temperature, blue blocks of the title compound were formed, collected and washed with dilute aqueous HCl.

### Refinement

All H atoms were placed in calculated positions, with C—H = 0.97 Å and N—H = 0.90 Å, and refined using a riding model, with *U*_iso_(H)=1.2*U*_eq_(C, N).

### Crystal data

**Table:**

(C_4_H_10_NO)_2_[CoCl_4_]	*F*(000) = 772
*M**_r_* = 376.99	*D*_x_ = 1.629 Mg m^−^^3^
Monoclinic, *P*2_1_/*c*	Mo *K*α radiation, λ = 0.71073 Å
Hall symbol: -P 2ybc	Cell parameters from 2761 reflections
*a* = 6.5952 (13) Å	θ = 2.5–26.0°
*b* = 13.696 (3) Å	µ = 1.80 mm^−^^1^
*c* = 17.039 (3) Å	*T* = 291 K
β = 92.930 (2)°	Block, blue
*V* = 1537.1 (5) Å^3^	0.26 × 0.12 × 0.08 mm
*Z* = 4	

### Data collection

**Table:**

Rigaku SCXmini diffractometer	2997 independent reflections
Radiation source: fine-focus sealed tube	2761 reflections with *I* > 2σ(*I*)
Graphite monochromator	*R*_int_ = 0.019
Detector resolution: 13.66612 pixels mm^-1^	θ_max_ = 26.0°, θ_min_ = 1.9°
ω scans	*h* = −7→8
Absorption correction: multi-scan (*CrystalClear*; Rigaku, 2005)	*k* = −16→16
*T*_min_ = 0.90, *T*_max_ = 1.00	*l* = −20→20
11708 measured reflections	

### Refinement

**Table:**

Refinement on *F*^2^	Primary atom site location: structure-invariant direct methods
Least-squares matrix: full	Secondary atom site location: difference Fourier map
*R*[*F*^2^ > 2σ(*F*^2^)] = 0.022	Hydrogen site location: inferred from neighbouring sites
*wR*(*F*^2^) = 0.074	H-atom parameters constrained
*S* = 1.07	*w* = 1/[σ^2^(*F*_o_^2^) + (0.055*P*)^2^ + 0.0459*P*] where *P* = (*F*_o_^2^ + 2*F*_c_^2^)/3
2997 reflections	(Δ/σ)_max_ = 0.001
154 parameters	Δρ_max_ = 0.25 e Å^−^^3^
0 restraints	Δρ_min_ = −0.42 e Å^−^^3^

### Special details

**Table:**

Geometry. All e.s.d.'s (except the e.s.d. in the dihedral angle between two l.s. planes) are estimated using the full covariance matrix. The cell e.s.d.'s are taken into account individually in the estimation of e.s.d.'s in distances, angles and torsion angles; correlations between e.s.d.'s in cell parameters are only used when they are defined by crystal symmetry. An approximate (isotropic) treatment of cell e.s.d.'s is used for estimating e.s.d.'s involving l.s. planes.
Refinement. Refinement of *F*^2^ against ALL reflections. The weighted *R*-factor *wR* and goodness of fit *S* are based on *F*^2^, conventional *R*-factors *R* are based on *F*, with *F* set to zero for negative *F*^2^. The threshold expression of *F*^2^ > σ(*F*^2^) is used only for calculating *R*-factors(gt) *etc*. and is not relevant to the choice of reflections for refinement. *R*-factors based on *F*^2^ are statistically about twice as large as those based on *F*, and *R*- factors based on ALL data will be even larger.

### Fractional atomic coordinates and isotropic or equivalent isotropic displacement parameters (Å^2^)

**Table:**

	*x*	*y*	*z*	*U*_iso_*/*U*_eq_	
C1	−0.1713 (3)	0.61037 (13)	0.55358 (11)	0.0444 (4)	
H1A	−0.2354	0.6639	0.5801	0.053*	
H1B	−0.2756	0.5629	0.5388	0.053*	
C2	−0.0788 (3)	0.64794 (13)	0.48120 (10)	0.0404 (4)	
H2A	−0.0210	0.5943	0.4526	0.048*	
H2B	−0.1819	0.6791	0.4472	0.048*	
C3	0.2348 (3)	0.67437 (14)	0.56019 (11)	0.0479 (4)	
H3A	0.3349	0.7226	0.5776	0.057*	
H3B	0.3043	0.6221	0.5342	0.057*	
C4	0.1303 (3)	0.63460 (14)	0.62962 (10)	0.0471 (4)	
H4A	0.2296	0.6033	0.6653	0.056*	
H4B	0.0696	0.6880	0.6576	0.056*	
C5	1.1478 (3)	0.07758 (13)	0.35868 (11)	0.0472 (4)	
H5A	1.1997	0.0142	0.3754	0.057*	
H5B	1.2597	0.1236	0.3616	0.057*	
C6	1.0622 (3)	0.07100 (13)	0.27501 (11)	0.0459 (4)	
H6A	1.0223	0.1355	0.2564	0.055*	
H6B	1.1652	0.0463	0.2415	0.055*	
C7	0.7310 (3)	0.03294 (13)	0.32823 (11)	0.0427 (4)	
H7A	0.6226	−0.0150	0.3280	0.051*	
H7B	0.6720	0.0957	0.3137	0.051*	
C8	0.8328 (3)	0.03920 (13)	0.40899 (10)	0.0428 (4)	
H8A	0.7346	0.0588	0.4464	0.051*	
H8B	0.8854	−0.0245	0.4245	0.051*	
Cl1	0.10686 (6)	0.80041 (3)	0.33040 (2)	0.04020 (12)	
Cl2	0.55058 (7)	0.83591 (3)	0.45501 (2)	0.04426 (13)	
Cl3	0.59472 (7)	0.79781 (4)	0.23183 (2)	0.04472 (13)	
Cl4	0.45151 (7)	0.59332 (3)	0.36450 (3)	0.04461 (13)	
Co1	0.44468 (3)	0.757637 (14)	0.342708 (12)	0.02945 (10)	
N1	0.0826 (2)	0.71964 (10)	0.50476 (8)	0.0392 (3)	
H1C	0.1438	0.7406	0.4618	0.047*	
H1D	0.0266	0.7716	0.5277	0.047*	
N2	0.8819 (2)	0.00444 (10)	0.27066 (8)	0.0395 (3)	
H2C	0.8231	0.0064	0.2218	0.047*	
H2D	0.9231	−0.0572	0.2804	0.047*	
O1	−0.02319 (19)	0.56588 (8)	0.60621 (7)	0.0402 (3)	
O2	0.9955 (2)	0.10839 (9)	0.40987 (7)	0.0454 (3)	

### Atomic displacement parameters (Å^2^)

**Table:**

	*U*^11^	*U*^22^	*U*^33^	*U*^12^	*U*^13^	*U*^23^
C1	0.0361 (9)	0.0437 (9)	0.0533 (11)	−0.0013 (7)	0.0020 (8)	0.0055 (8)
C2	0.0476 (10)	0.0389 (8)	0.0335 (9)	0.0042 (7)	−0.0083 (7)	−0.0026 (7)
C3	0.0440 (10)	0.0619 (11)	0.0373 (9)	−0.0188 (8)	−0.0023 (8)	0.0045 (8)
C4	0.0601 (11)	0.0511 (10)	0.0292 (9)	−0.0191 (9)	−0.0060 (8)	0.0041 (7)
C5	0.0418 (10)	0.0453 (9)	0.0544 (11)	−0.0079 (8)	0.0031 (8)	−0.0097 (8)
C6	0.0550 (11)	0.0393 (8)	0.0448 (10)	−0.0053 (8)	0.0159 (8)	0.0002 (8)
C7	0.0412 (9)	0.0418 (9)	0.0450 (10)	−0.0019 (7)	0.0015 (8)	−0.0046 (7)
C8	0.0515 (10)	0.0412 (9)	0.0363 (9)	−0.0102 (7)	0.0061 (8)	−0.0041 (7)
Cl1	0.0353 (2)	0.0502 (2)	0.0352 (2)	0.00980 (16)	0.00288 (17)	0.01069 (17)
Cl2	0.0559 (3)	0.0427 (2)	0.0342 (2)	−0.01302 (18)	0.00315 (19)	−0.00842 (17)
Cl3	0.0403 (2)	0.0616 (3)	0.0330 (2)	−0.00275 (18)	0.00952 (18)	0.00554 (18)
Cl4	0.0543 (3)	0.0295 (2)	0.0487 (3)	−0.00623 (16)	−0.0107 (2)	0.00300 (16)
Co1	0.03183 (15)	0.02976 (14)	0.02686 (15)	−0.00168 (7)	0.00255 (10)	0.00081 (7)
N1	0.0550 (9)	0.0368 (7)	0.0266 (7)	−0.0064 (6)	0.0108 (6)	0.0019 (6)
N2	0.0549 (9)	0.0347 (7)	0.0283 (7)	0.0001 (6)	−0.0038 (6)	−0.0015 (5)
O1	0.0462 (7)	0.0356 (6)	0.0384 (6)	−0.0088 (5)	−0.0005 (5)	0.0075 (5)
O2	0.0522 (7)	0.0415 (6)	0.0428 (7)	−0.0124 (6)	0.0053 (6)	−0.0148 (5)

### Geometric parameters (Å, º)

**Table:**

C1—O1	1.428 (2)	C6—N2	1.497 (2)
C1—C2	1.495 (2)	C6—H6A	0.9700
C1—H1A	0.9700	C6—H6B	0.9700
C1—H1B	0.9700	C7—N2	1.485 (2)
C2—N1	1.488 (2)	C7—C8	1.503 (2)
C2—H2A	0.9700	C7—H7A	0.9700
C2—H2B	0.9700	C7—H7B	0.9700
C3—N1	1.479 (2)	C8—O2	1.431 (2)
C3—C4	1.502 (2)	C8—H8A	0.9700
C3—H3A	0.9700	C8—H8B	0.9700
C3—H3B	0.9700	Co1—Cl1	2.3029 (6)
C4—O1	1.424 (2)	Co1—Cl2	2.2720 (6)
C4—H4A	0.9700	Co1—Cl3	2.2455 (6)
C4—H4B	0.9700	Co1—Cl4	2.2811 (6)
C5—O2	1.427 (2)	N1—H1C	0.9000
C5—C6	1.509 (3)	N1—H1D	0.9000
C5—H5A	0.9700	N2—H2C	0.9000
C5—H5B	0.9700	N2—H2D	0.9000
			
O1—C1—C2	111.70 (14)	C5—C6—H6B	109.7
O1—C1—H1A	109.3	H6A—C6—H6B	108.2
C2—C1—H1A	109.3	N2—C7—C8	109.66 (14)
O1—C1—H1B	109.3	N2—C7—H7A	109.7
C2—C1—H1B	109.3	C8—C7—H7A	109.7
H1A—C1—H1B	107.9	N2—C7—H7B	109.7
N1—C2—C1	108.73 (14)	C8—C7—H7B	109.7
N1—C2—H2A	109.9	H7A—C7—H7B	108.2
C1—C2—H2A	109.9	O2—C8—C7	110.32 (14)
N1—C2—H2B	109.9	O2—C8—H8A	109.6
C1—C2—H2B	109.9	C7—C8—H8A	109.6
H2A—C2—H2B	108.3	O2—C8—H8B	109.6
N1—C3—C4	109.34 (15)	C7—C8—H8B	109.6
N1—C3—H3A	109.8	H8A—C8—H8B	108.1
C4—C3—H3A	109.8	Cl3—Co1—Cl2	117.59 (2)
N1—C3—H3B	109.8	Cl3—Co1—Cl4	111.892 (19)
C4—C3—H3B	109.8	Cl2—Co1—Cl4	109.01 (2)
H3A—C3—H3B	108.3	Cl3—Co1—Cl1	109.083 (19)
O1—C4—C3	111.56 (14)	Cl2—Co1—Cl1	102.183 (19)
O1—C4—H4A	109.3	Cl4—Co1—Cl1	106.068 (18)
C3—C4—H4A	109.3	C3—N1—C2	110.39 (13)
O1—C4—H4B	109.3	C3—N1—H1C	109.6
C3—C4—H4B	109.3	C2—N1—H1C	109.6
H4A—C4—H4B	108.0	C3—N1—H1D	109.6
O2—C5—C6	110.74 (15)	C2—N1—H1D	109.6
O2—C5—H5A	109.5	H1C—N1—H1D	108.1
C6—C5—H5A	109.5	C7—N2—C6	111.39 (13)
O2—C5—H5B	109.5	C7—N2—H2C	109.3
C6—C5—H5B	109.5	C6—N2—H2C	109.3
H5A—C5—H5B	108.1	C7—N2—H2D	109.3
N2—C6—C5	109.93 (14)	C6—N2—H2D	109.3
N2—C6—H6A	109.7	H2C—N2—H2D	108.0
C5—C6—H6A	109.7	C4—O1—C1	110.33 (13)
N2—C6—H6B	109.7	C5—O2—C8	110.38 (12)
			
O1—C1—C2—N1	−58.22 (18)	C8—C7—N2—C6	−53.66 (19)
N1—C3—C4—O1	57.4 (2)	C5—C6—N2—C7	52.48 (19)
O2—C5—C6—N2	−55.95 (19)	C3—C4—O1—C1	−58.9 (2)
N2—C7—C8—O2	58.34 (18)	C2—C1—O1—C4	59.74 (19)
C4—C3—N1—C2	−56.12 (19)	C6—C5—O2—C8	61.60 (19)
C1—C2—N1—C3	56.46 (18)	C7—C8—O2—C5	−62.79 (19)

### Hydrogen-bond geometry (Å, º)

**Table:**

*D*—H···*A*	*D*—H	H···*A*	*D*···*A*	*D*—H···*A*
N1—H1*C*···Cl1	0.90	2.39	3.1819 (15)	148
N1—H1*D*···O2^i^	0.90	1.97	2.8294 (19)	160
N2—H2*C*···O1^ii^	0.90	2.47	3.0577 (18)	123
N2—H2*C*···Cl4^iii^	0.90	2.57	3.3322 (15)	143
N2—H2*D*···Cl1^iv^	0.90	2.43	3.3003 (15)	164

Symmetry codes: (i) −*x*+1, −*y*+1, −*z*+1; (ii) *x*+1, −*y*+1/2, *z*−1/2; (iii) −*x*+1, *y*−1/2, −*z*+1/2; (iv) *x*+1, *y*−1, *z*.
